# Graphene-coated meshes for electroactive flow control devices utilizing two antagonistic functions of repellency and permeability

**DOI:** 10.1038/ncomms13345

**Published:** 2016-10-31

**Authors:** Rassoul Tabassian, Jung-Hwan Oh, Sooyeun Kim, Donggyu Kim, Seunghwa Ryu, Seung-Min Cho, Nikhil Koratkar, Il-Kwon Oh

**Affiliations:** 1 Creative Research Initiative Center for Functionally Antagonistic Nano-Engineering, Department of Mechanical Engineering, Korea Advanced Institute of Science and Technology (KAIST), 291 Daehak-ro, Yuseong-gu, Daejeon 34141, Republic of Korea; 2 Department of Mechanical Engineering, Korea Advanced Institute of Science and Technology (KAIST), 291 Daehak-ro, Yuseong-gu, Daejeon 34141, Republic of Korea; 3 New Business Division, Hanwha Techwin R&D Center, 6, Pangyo-ro 319beon-gil, Bundang-gu, Seongnam-si, Gyeonggi-do 13488, Republic of Korea; 4 Department of Mechanical, Aerospace and Nuclear Engineering, Rensselaer Polytechnic Institute, 110 8th Street, Troy, New York 12180, USA; 5 Department of Materials Science and Engineering, Rensselaer Polytechnic Institute, 110 8th Street, Troy, New York 12180, USA

## Abstract

The wettability of graphene on various substrates has been intensively investigated for practical applications including surgical and medical tools, textiles, water harvesting, self-cleaning, oil spill removal and microfluidic devices. However, most previous studies have been limited to investigating the intrinsic and passive wettability of graphene and graphene hybrid composites. Here, we report the electrowetting of graphene-coated metal meshes for use as electroactive flow control devices, utilizing two antagonistic functions, hydrophobic repellency versus liquid permeability. Graphene coating was able to prevent the thermal oxidation and corrosion problems that plague unprotected metal meshes, while also maintaining its hydrophobicity. The shapes of liquid droplets and the degree of water penetration through the graphene-coated meshes were controlled by electrical stimuli based on the functional control of hydrophobic repellency and liquid permeability. Furthermore, using the graphene-coated metal meshes, we developed two active flow devices demonstrating the dynamic locomotion of water droplets and electroactive flow switching.

## Abstract (web summary)

The wettability properties of graphene hold promise for the realisation of flow control devices. Here, the authors demonstrate that the degree of water penetration through a nickel mesh coated with graphene can be controlled electrically, enabling dynamic locomotion of water droplets.

Graphene has attracted a considerable amount of attention in a variety of academic and industrial fields due to its superior material properties, including its high thermal and electrical conductivities, good chemical stability, flexibility, optical transparency, large surface areas, and mechanical stiffness and strength^1^,^2^,^3^,^4^,^5^,^6^,^7^,^8^,^9^,^10^,^11^,^12^,^13^,^14^,^15^,^16^,^17^,^18^,^19^,^20^. Interestingly, recent progress reveals that monolayer graphene is wetting-transparent^21^,^22^,^23^ to an underlying substrate provided that the substrate is significantly more wettable (hydrophilic) than the graphene. In fact, the wetting transparency effect can also be extended to strongly hydrophobic (that is, rough) substrates^24^, if the graphene coating is deposited *in-situ* via chemical vapour deposition (CVD) and conforms to the surface roughness features of the substrate. These results indicate that graphene-coated metal substrates can be used as water harvesting devices with greatly improved anti-oxidation and anti-corrosion capabilities, without disrupting the intrinsic wettability of the underlying substrate^21^,^22^,^23^,^24^,^25^. However, previous studies only investigated the passive wettability of graphene and graphene hybrid composites; to the best of our knowledge, active control of the wettability of graphene-coated surfaces has not been investigated thus far. In this work, the wettability of graphene-coated surfaces is extended to electrowetting and electroactive flow devices, which could lead to a variety of practical applications in the areas of microfluidics and nanofluidics.

Conventionally, mesh structures, which consist of woven strands of metal, fibre, wire and other ductile materials, have been widely used as filters to remove impurities or to recover solids from various liquid flow systems due to their liquid permeability through their pores. By physically tailoring the pore size and weave shape, and chemically modifying the surfaces, metal meshes were used as a substrate for hydrophobic and oleophilic surfaces in pioneering works^26^,^27^,^28^. Most interestingly, when water droplets are placed on a hydrophobic fine mesh, they do not penetrate through the mesh due to the resistance of capillary forces arising in the voids of the mesh, resulting in hydrophobic repellency. However, water droplets are able to pass through the mesh, when the external pressure or another force is large enough to exceed the capillary force^29^,^30^. Therefore, active flow control devices driven by the electrostatic potential can be realized by properly tailoring two mutually antagonistic functions, that is, liquid permeability versus hydrophobic repellency. If the thermal oxidation and corrosion problems that frequently occur during the process of electrowetting could be solved, highly conductive metal mesh structures could be used as active flow control devices in a manner analogous to the electrical switches and transistors in electronic circuits. Graphene coating onto metal mesh structures is a promising approach to protect against corrosion and oxidation. It can also lead to mechanically durable hydrophobic surfaces with accurately preservation of other beneficial properties of the mesh structure, such as the porosity, roughness and hydrophobicity^31^,^32^.

Here, we report the electrowetting of graphene-coated metal meshes as a part of the effort to develop electroactive flow control devices utilizing two antagonistic functions of liquid permeability and hydrophobic repellency. Graphene was uniformly coated onto a nickel (Ni) mesh by means of CVD. The shape of a liquid droplet and the degree of water penetration through the graphene-coated meshes were controlled by electrical stimuli, which was explained in terms of the free energy change. Furthermore, we demonstrated two types of active flow devices using graphene-coated metal meshes: the dynamic locomotion of water droplets between layered electrodes and electroactive flow switching devices. These results highlight the possibility of using graphene-coated porous structures in active flow control applications.

## Results

### Synthesis and characterization of graphene-coated nickel mesh

**Figure 1:**
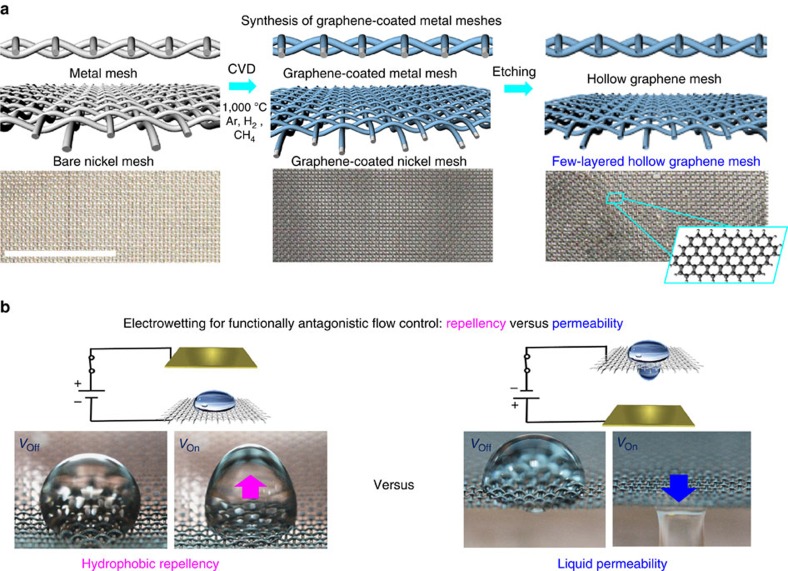
Schematic of synthetic route to graphene-coated metal mesh and hollow graphene mesh. (**a**) Fabrication steps of graphene-coated metal meshes. Scale bar, 1 cm. (**b**) Electrowetting and functionally antagonistic flow control using liquid repellency and liquid permeability.

The most effective route to the conformal and uniform deposition of graphene layers on metal meshes is to use the bottom–up CVD approach. Graphene grown by the CVD method has a continuous and monolithic surface, and exhibits enhanced electrical and mechanical properties due to the strong bonding between graphene micro-sheets and the low concentration of defects^8^. Figure 1a shows a schematic for the synthesis of a graphene-coated nickel mesh (GCNM). In this study, nickel (Ni) wire woven meshes were used as a catalytic substrate, which is necessary to grow few-layered graphene, as described in the Supplementary Note 1 and shown in Supplementary Figs 1 and 2. Graphene growth was realized by heating the Ni mesh substrates up to 1,000 °C and flowing methane gas as a carbon source^11^,^14^. The graphene coating on the Ni mesh slightly changed the colour of the mesh from silver to grey due to the multiple graphene layers. Free-standing, few-layered and hollow graphene meshes were obtained after an etching process and very good continuity and integrity of the graphene meshes were observed, as shown in the following scanning electron microscope (SEM) and transmission electron microscopy images.

The prepared GCNM was then utilized as an electrode for the electrical stimulation of a water droplet. Figure 1b presents the control of the repellency and permeability of liquid droplets on the GCNM by electrical stimuli. 10 mm × 20 mm GCNM and a Cu tape electrode were used as the negative and positive electrodes, respectively. The two antagonistic wetting effects of liquid permeability and hydrophobic repellency were controlled by electric stimuli. These effects will be discussed in detail in the following sections.

**Figure 2:**
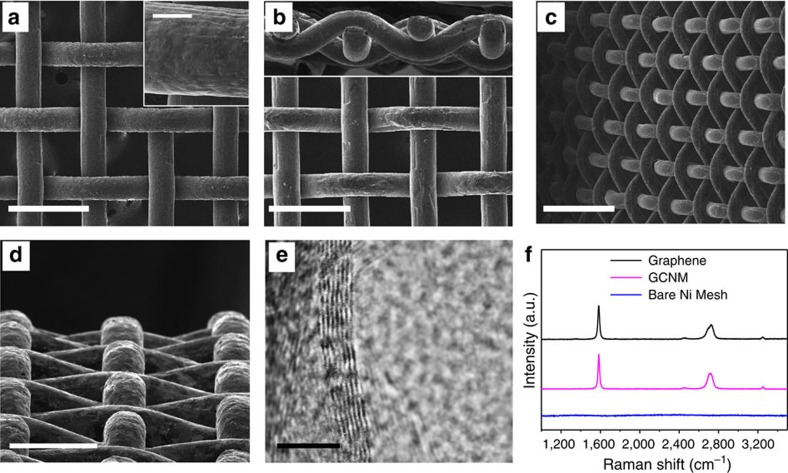
SEM and transmission electron microscopy (TEM) images of graphene-coated metal mesh and hollow graphene mesh. (**a**) Graphene coated on Ni mesh. Scale bar, 300 μm. Inset is a closer view of graphene coating on metal mesh. Inset scale bar, 40 μm. (**b**,**c**,**d**) Pure graphene mesh after etching process. Scale bars are 300, 500 and 200 μm, respectively. (**e**) TEM image of multilayer CVD-grown graphene. Scale bare, 5 nm. (**f**) Raman spectra of GCNM, bare nickel mesh, and hollow graphene mesh after etching of GCNM.

The morphology of the GCNM was carefully investigated by SEM images, as shown in Fig. 2a. The few-layered graphenes fully covered the Ni meshes without void areas; this is very important with regard to the anti-corrosion and anti-oxidation of metal meshes. After the growth of the graphene layers, the etching process of Ni skeleton was simply performed by soaking the GCNM in a 3 M HCl solution for 24 h to examine the quality of the hollow graphene structure, as described in Supplementary Note 2 and Supplementary Fig. 3. The pure tubular graphene mesh can be obtained after freeze-drying process as shown in Supplementary Fig. 4. It was observed that tubular graphene sheets were woven with each other and that the interval distances of the pure graphene mesh were identical to that of the original Ni mesh template (Fig. 2b–d). The number of graphene layers is a critical factor, which allows the hollow graphene meshes to retain their free-standing structural shape. The number of the CVD-grown graphene layers was carefully investigated using transmission electron microscopy images. It revealed that the number of layers varied between 4 and 7 as shown in Fig. 2e. Multiple graphene layers prevent the formation of cracks and voids on the coating surface, which significantly enhanced the protective roles of graphene against corrosion and thermal oxidation.

Figure 2f presents the Raman spectra of three different samples (bare Ni mesh, GCNM and Ni-etched graphene). The intensity ratio of the 2D peak (at 2,750 cm^−1^) and the G peak (at 1,580 cm^−1^) is related to the number of graphene layers. The Raman spectra of graphene layers exhibit a higher intensity of the G peaks to the 2D peaks, indicating a multilayered graphene structure^33^,^34^. Moreover, the absence of a defect-induced D peak (between 1,270 and 1,450 cm^−1^) indicates that high-quality graphene was coated onto the mesh with low-defect concentrations^35^.

Generally high-quality graphene coating can increase the degree of resistance to oxidation and corrosion while retaining the wetting properties. The measurement of the contact angle (CA) was conducted at four different points of the samples to investigate the effect of the graphene coating on the wetting properties of the GCNM. The CA of water droplets on both bare Ni mesh and GCNM are nearly identical, as shown in Supplementary Fig. 5, demonstrating the wetting transparency^21^,^22^,^23^,^24^,^25^ of the graphene coating on the metal substrates. To evaluate the protective effect of graphene against thermal oxidation, we thermally annealed both GCNM and bare Ni mesh at 400 °C for 4 h. After the thermal annealing process, the colour of the bare Ni mesh significantly changed from silver to dark blue, whereas the colour of the graphene-coated samples remained unchanged. The X-ray photoelectron spectroscopy data of the bare Ni mesh after thermal oxidation shows a remarkable increase in the intensity of the NiO peak, compared with that of the bare Ni mesh before thermal oxidation (Supplementary Fig. 6a,b). Unlike the bare Ni mesh, the X-ray photoelectron spectroscopy data of the GCNM presents weak red and blue peaks corresponding to C=O and C–O bonds, respectively. Thermal oxidation significantly affects the wettability of the bare Ni mesh, switching its surface from hydrophobic to hydrophilic as the exposure time elapses. The CA of the water droplets on the bare Ni mesh sharply decreases during thermal oxidation. As a result, water droplets penetrate into the GCNM with longer than 5 min of the oxidation time. However, the CA of the GCNM remained unchanged after thermal annealing, indicating that the graphene coating against thermal oxidation plays a robust protective role (Supplementary Fig. 6c). The graphene coating also showed a protective effect against the chemical reactions associated with corrosion processes. The cyclic voltammetry response of specimens in KOH solution (1 M) with a scan rate of 100 mV s^−1^ shows that GCNM has negligible oxidation and reduction peaks as compared with the bare Ni samples (Supplementary Fig. 6d). These results indicate that the graphene coating acts as a barrier against electrochemical reactions and significantly decreases the oxidation and reduction rates.

### Electrowetting of graphene-coated nickel mesh

For a better understanding of the hydrophobic repellency and liquid permeability, the wetting state of the mesh must be comprehended clearly. The GCNM has a unique rough surface with periodic topographical feature, which makes it different from conventional flat surfaces. Depending on the wetting states of the mesh, Wenzel or Cassie models can be considered^29^. In our case, the GCNM showed Cassie state. When a water droplet was placed on the mesh, it remained stable without penetration inside the mesh (hydrophobic repellency), due to the negative capillary effect originated from liquid–air interface in the mesh openings. We can approximate the Laplace pressure produced by the liquid–air interface in the mesh openings as follows (See more details in the Supplementary Note 3).


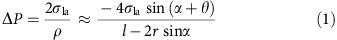


where *σ*_la_ is the surface tension of the liquid–air interface, *ρ* is the curvature radius of the liquid and *θ* is the intrinsic CA of the liquid on the flat graphene-covered Ni surface. *r* and *l* are the radius of each wire and the distance between the centres of the wires, respectively (Supplementary Fig. 7). When the droplet touches the mesh, *α* increases until Δ*P* becomes equal to the pressure induced by gravity or other external forces. To change wetting states from the Cassie state to the Wenzel state, that is, to force the drop to pass the mesh (liquid permeability), the pressure induced by external forces must exceed the maximum value of Δ*P* in equation (1). We found that, for the GCNM considered in our study, a very large electric field around 33 kV cm^−1^ is required to overcome the Laplace pressure by Maxwell stress, and the threshold electric field is inversely proportional to 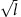 (See the Supplementary Note 3 for detail). Hence, unless another droplet is placed on the opposite side of the mesh, liquid permeation is practically prohibited. On the other hand, the hydrophobicity of the GCNM is easily tunable by *l* and *r*; the CA rises with an increase in the fraction of the air–liquid interface (that is, larger *l* or smaller *r*), and vice versa.

**Figure 3:**
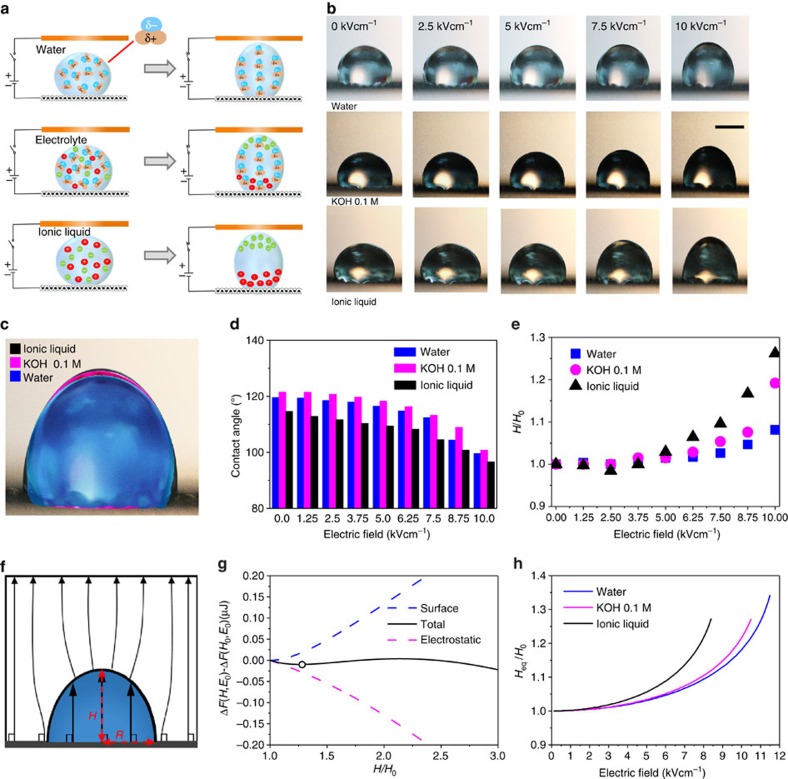
Electrostatic actuation of liquid droplets under electric fields. (**a**) Shape control of three different liquids by electric field. (**b**) Optical images of liquid droplets (water, KOH 0.1 M and ionic liquid) under different electric fields. Scale bark, 1 mm. (**c**) Comparison of shape changes of three liquid droplets under electric field of 10 kV cm^−1^. (**d**) Changes of CA under different electric fields. (**e**) Variation of normalized height under electric fields. (**f**) Electric field flux when CA=90°. Under this condition, the electric field becomes uniform inside the droplet. (**g**) The free energy change as a function of droplet height under an applied electric field of *E*_0_=11 kV cm^−1^ (solid line). Surface and electrostatic contributions are represented by dashed lines. The equilibrium droplet height is suitably predicted from the local minimum (marked by white circle). (**h**) The droplet height as a function of the applied electric field. Beyond the critical electric field, the droplet height prediction diverges, indicating the instability of the droplet.

The actuation of different liquid droplets was studied by placing droplets under an electric field as illustrated in Fig. 3b. Distilled (DI) water, 0.1 M KOH aqueous solution and ionic liquid (1-Butyl-3-methylimidazolium dicyanamide) were used as liquid droplets, with similar volumes (5 μl). They were placed on the GCNM as a negative electrode while a Cu tape electrode was used as a positive electrode. The electric field results in shape changes of the liquid droplets due to the dielectric alignment of the polar water molecules and the migrations of dissociated ions (Fig. 3a)^36^. Dissociated anions moved towards the cathode electrode, while cations moved towards the anode electrode under an applied electric field.

**Figure 4:**
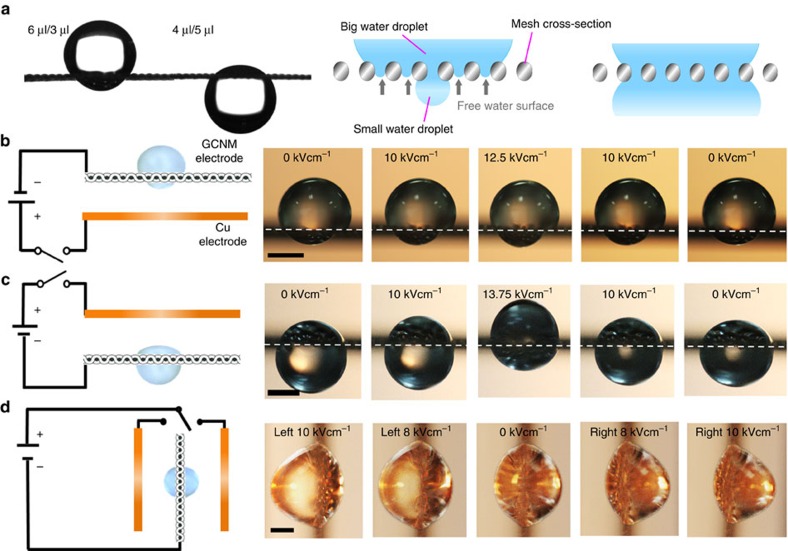
Reversible actuation modes by electrowetting of graphene-coated Ni meshes. (**a**) Comparison of stationary droplet shapes when placing two differently sized droplets on both sides of the mesh and mechanism of penetration based on capillary forces in mesh openings. (**b**–**d**) Reversible actuation modes of two water droplets placed on both sides of the mesh with different electrode combinations. Scale bar, 1 mm.

When applying an electric field up to 10 kV cm^−1^, all three liquid droplets were stretched towards the Cu tape electrode and the overall shapes of the droplets changed, as shown in Fig. 3b,c. While the drop stretched upward, the CA also changed, decreasing from 119.2° to 103.2° for the water, from 121.5° to 100.8° for the KOH solution and from 114.6° to 96.6° for the ionic liquid (Fig. 4d). Figure 3e clarifies the shape change of the three liquid droplets under different electric fields by plotting the variation of the normalized height of each droplet. The height of the water, KOH and ionic liquids under 10 kV cm^−1^ electric field increased up to 8.1, 16.6 and 26.2%, respectively. The changes in the height and CA for the water droplet are relatively small compared with those of the KOH solution and the ionic liquid, indicating that the electrostatic force strongly influences the ion movements in the KOH solution and ionic liquid.

The change of the droplet shape can be understood in terms of the free energy change. In the presence of a uniform external electric field **E**_0_, the droplet insertion free energy can be expressed as follows (See the Supplementary Note 4 for details)^37^,^38^,^39^,


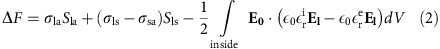


where, *σ*_ls_ and *σ*_sa_ represent the surface energies of the liquid-substrate and substrate-air, respectively. *S*_la_ and *S*_ls_ are correspondingly the surface areas of the droplet in contact with air and the substrate. 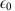 is the permittivity in a vacuum, while 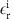 and 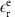 are the relative permittivity of liquid and air. **E**_**1**_ is the electric field inside the droplet and the integral is performed within the droplet volume. The first two terms are associated with the surface energy of the droplet, and the third term corresponds to the electrostatic free energy of the system. The free energy change, Δ*F*, is analytically solvable for the case of CA=90° because the electric field inside the droplet becomes uniform due to the unique boundary condition, as depicted in Fig. 3f. In addition, we derive the closed-form expression. (See the Supplementary Note 4 for details)


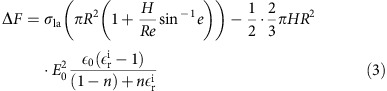


where, *H* and *R* are the long and short semi-principal axes of the droplet with the half prolate ellipsoidal shape (see Fig. 3f), 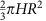 is the volume of the droplet, 
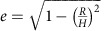
 is the eccentricity of the ellipsoid and the geometrical factor is given as a function of the eccentricity, as 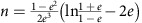. While the CAs in our experiments are not 90°, the analytic solution for 90° provides a qualitative understanding of the underlying physics behind electrowetting without concerning computationally expensive numerical solutions for general CAs.

Given that the volume of the droplet is constant, we can express Δ*F* (*H*, *E*_0_) as a function of two variables, *H* and *E*_0_. In the theoretical calculations, we use a water surface tension of *σ*_la_=72 mNm^−1^, an ionic liquid surface tension of *σ*_la_=46 mNm^−1^ 40, relative permittivity of water of 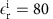, and a droplet volume of 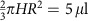. Given the applied electric field *E*_0_, we can find the droplet height *E*_eq_ at the local free energy minimum, as shown in Fig. 3g for the water droplet under *E*_0_=11 kV cm^−1^. By repeating the calculation with a wide range of *E*_0_, we can predict the droplet height as a function of the applied electric field, as depicted in Fig. 3h. The responses of the distilled water, the KOH solution and the ionic liquid differ owing to their different surface tensions and relative permittivity factors. The predictions of the conducting objects such as the droplets of KOH solution and ionic liquid, can be obtained at the limit of the infinite relative permittivity, that is, 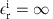 (ref. 39).

The theoretical predictions are consistent with the experimental results in that the droplet height increases nonlinearly with the applied electric field, and that the difference between the distilled water and the KOH solution is relatively small, whereas the ionic liquid shows significantly higher sensitivity to the applied electric field. The predicted droplet height *H*_eq_ diverges beyond the critical electric field, above which the local minimum in Fig. 3g disappears (See the additional details in the Supplementary Note 6). In experiments, droplets break and split into a few pieces beyond critical electric field due to instability. Because the last term in Δ*F* (*H*, *E*_0_) becomes scarcely sensitive to 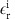 in the range of 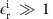, the predictions for distilled water and KOH solution lie within a small interval, as shown in Fig. 3f–h. The ionic liquid droplet is more sensitive to the applied electric field, due to its low surface tension.

Further experiments to assess the degree of liquid permeability by electrowetting were conducted with water droplets placed on the GCNM. For these tests, we placed water drops on both the upper and the lower surfaces of the GCNM to reduce the threshold electric field for the permeation. When the water droplet on the top of the mesh was much larger than that underneath the mesh, the larger droplet stayed on the mesh (Fig. 4a). In this case, there are numerous empty holes below the upper drop, which are not still occupied by water molecules. As discussed earlier, the negative capillary effect generated in these holes resists against the penetration of the upper droplets. This causes the upside droplet to remain on top of the mesh. On the other hand, when the size of the droplet on the top of the mesh is equal or smaller than that of underneath, the upper drop easily passes through the mesh by gravity, as all holes are filled with water molecules and no capillary effect is generated. In this case, the upper drop will merge with the droplet underneath the mesh, forming a much larger stationary droplet below the GCNM. The flow of water through the porous mesh structure is dictated by the balance of the capillary forces and gravitational force.

Figure 4b–d show the reversible actuation modes of two droplets placed on both the upper and lower surfaces under various electrical fields. The droplet tends to move toward the side under the applied electric field to lower the electrostatic free energy of the system, and the asymmetry is caused by the effect of gravitational force. We note that the free energy change is a monotonically decreasing function of the applied electric field *E*_0_ (Supplementary Fig. 8); hence, it is always favourable to have the droplet in the side under the electric field. However, the droplet locomotion through the mesh only occurs when the electric field is large enough to cause a significant change of the height for a single droplet with a similar volume. As shown in Fig. 4b, the shape changes of the large and small droplets placed on the upper and lower surfaces can be reversibly and controllably adjusted by applying electric voltage. When an electric field is applied under the GCNM, the shape of the smaller droplet underneath the mesh changes. The larger droplet remains on the mesh due to hydrophobic repellency, while the smaller drop is more easily affected by the applied field, with its shape and CA both changing. In another set-up, as shown in Fig. 4c, one droplet (5 μl) was placed on the mesh, while another droplet of the same size was positioned underneath the mesh. A Cu tape electrode was located above the mesh. By applying an electric field up to 13.75 kV cm^−1^, the droplet underneath the mesh moved to the upper droplet through the mesh, and the fused droplets moved towards the Cu electrode. When the applied voltage was decreased to 0 kV cm^−1^, the droplets returned to their original shapes. In this case, because the sizes of both drops are equal, the applied bias input voltage is sufficient to drive the lower drop towards the upper drop, and the effect is reversed when the applied bias is removed. However, if one drop is much larger than the other, the larger drop remains unaffected (as shown in Fig. 4b), while only the smaller drop is affected by the applied voltage. The Supplementary Movie 1 shows this actuation in greater detail.

Furthermore, to avoid the effects of the gravitational force, we tested a new vertical electrode set-up with two Cu and one GCNM electrodes as shown in Fig. 4d. Two droplets of identical sizes were placed on both sides of the GCNM. By applying and switching the electric fields, dynamic and reversible horizontal motion of the droplets occurred through the mesh. This design with two droplets of identical sizes can be used to control the shapes of droplets by adjusting only the electrostatic force, as no gravity force affects the actuation.

**Figure 5:**
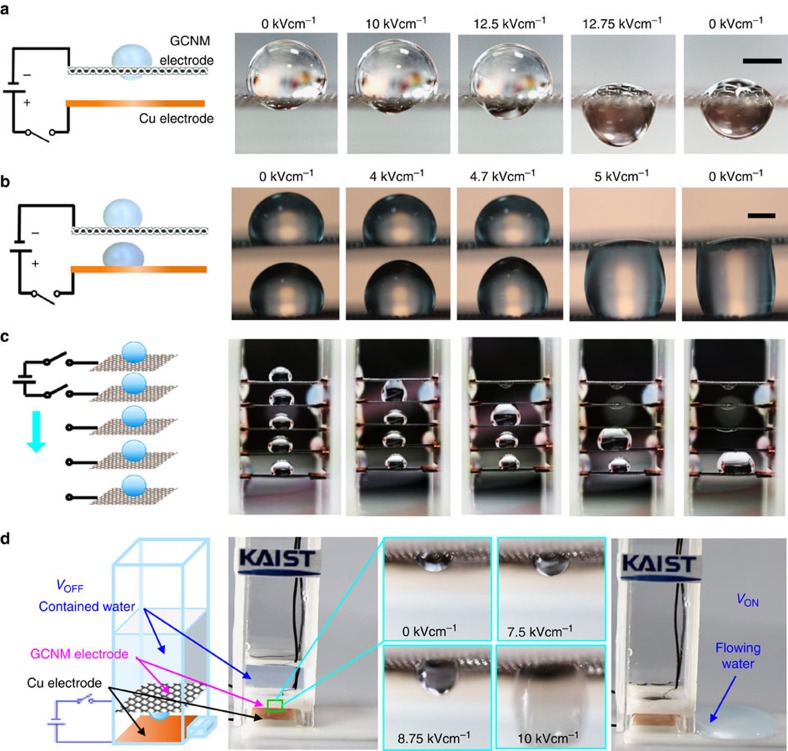
Irreversible actuation modes and functionally antagonistic active flow devices. (**a**) Irreversible actuation of water droplets on both sides of the mesh. Scale bar, 1 mm. (**b**) Irreversible actuation of two water droplets placed on different electrodes. Scale bar, 1 mm. (**c**) Locomotion of water droplets on multi-stage GCNM electrodes, and (**d**) Electroactive flow switching device based on GCNM.

In addition to the aforementioned reversible actuation modes, we observed irreversible actuations at higher applied voltage levels. As an example, Fig. 5a shows a higher applied voltage level with a configuration identical to that in Fig. 4b. Beyond an applied electric field of 12.5 kV cm^−1^, the electrostatic force pulls the larger (top) drop through the mesh and merges the two drops. When the combined electrostatic and gravitational force becomes large enough, the larger drop irreversibly passes through the mesh. The permeation threshold of the electric field can be estimated by comparing the free energies of two states; the first state with droplets on both sides of meshes and the second state with one droplet at the top of the mesh and the other free elliptical droplet below (See the Supplementary Note 4 and Supplementary Fig. 9). After the larger droplet penetrates into the GCNM under an electric potential of 12.75 kV cm^−1^, it cannot return to its original (initial) state when the applied electric potential is removed. Next, we designed a novel electrode system with two water droplets and two electrodes, with one droplet per electrode, respectively, as shown in Fig. 5b. When an electrical field of 5 kV cm^−1^ was applied to the electrode system, the lower water droplet on the Cu electrode began stretching towards the GCNM, while the shape of the upper water droplet on the GCNM did not change in its shape. However, when the lower water droplet came into contact with the GCNM electrode at 5 kV cm^−1^, the upper droplet abruptly passed through the GCNM mesh, as shown in Fig. 5b, due to the attractive and cohesive forces between the water drops. The electric field within the gap between the mesh and top of the droplet is greatly magnified (See Supplementary Note 5 and Supplementary Fig. 10 for details), which cause the droplet at the bottom to touch the mesh on the upper size under a relatively small electric field. When the applied electric field was turned off, the merged water drop maintains this state without returning to the original states of the individual drops, thus showing an irreversible actuation mode. The Supplementary Movie 2 shows this in detail. The divergence of the droplet behaviour under electric fields was described in Supplementary Note 6 and free energy as a function of the droplet height for a 5 μl droplet at three electric fields is shown in Supplementary Fig. 11.

## Discussion

The aforementioned actuation modes of water droplets on the mesh can be expanded to practical engineering applications. Here, we propose two flow control devices that take advantages of the intelligent penetration of water droplets through the GCNM. In the first design, the dynamic movement of water droplets through multi-stage mesh electrodes was demonstrated as shown in Fig. 5c. A series of parallel mesh electrodes were aligned in the vertical direction, and water droplets were placed on each mesh. When an electrical field was applied to the first and second GCNM electrodes, the water droplet between them was actuated upward and suddenly pulled down the droplet placed on the upper electrode. The actuation mechanism is identical to that discussed in Fig. 5b. The highest water droplet merged into the second water droplet after passing through the first GCNM. Switching the location of the applied voltage between the two mesh electrodes sequentially caused the movement of water droplets from the top mesh to the bottom mesh electrodes, as shown in Fig. 5c and in Supplementary Movie 3. Finally, all combined droplets were accumulated on the bottom mesh electrode. This demonstration suggests a potential for microfluidics applications to control the dynamic movement and combination of liquid droplets.

The second design was an electroactive flow switcher, which functionally utilized antagonistic control between hydrophobic repellency and liquid permeability, as presented in Fig. 5d. A GCNM electrode and a Cu electrode were installed inside a rectangular flow channel standing vertically. In the absence of an electrical field, water was contained above the GCNM electrode without passing through the mesh due to the hydrophobic repellency of the mesh itself. When the electrical field was increased beyond a critical voltage, the contained water passed through the GCNM (Supplementary Movie 4). To the best of our knowledge, this is the first demonstration of an electroactive flow switch based on graphene-coated metal meshes.

We note that the critical electric field for droplet instability (the divergence of the height) scales as 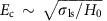 (see the Supplementary Notes 5 and 6). For example, a larger electric field by ten times is required to induce the instability for a drop smaller by 100 times. In comparison, because the applied electric field scales linearly with the inverse of the electrode distance, a larger electric field by 100 times can be applied with the same voltage. Hence, if the entire system is scaled down by 100 times, we can induce an identical degree of change in the droplet shape by making the applied voltage smaller by 10 times. This suggests the feasibility of applying the technique suggested here to a variety of micro/nanofluidic machinery with much smaller driving voltage. We note that there exists a potential limitation with regard to the scaled-down experiments because the actuation mechanism is based on the electrostatically driven free energy change, formulated by equation (3). The continuum description would not work when the droplet size is down to a few nanometres.

In summary, we report the electrowetting of graphene-coated metal meshes to produce active flow control devices by electrically tailoring two antagonistic functions: hydrophobic repellency versus liquid permeability. A graphene coating on metal meshes can resolve the critical oxidation and corrosion problems which occur during the process of the electrowetting of metal meshes, and it is beneficial when attempting to produce mechanically durable hydrophobic surfaces. The present results show that electric stimuli on graphene-coated metal meshes can be used to control the shapes of liquid droplets, as well as the CA, hydrophobic repellency and dynamic movements of water droplets. Moreover, we demonstrated two types of active flow devices to produce the dynamic locomotion of water droplets between layered electrodes and electroactive flow switching. As a further study, in-depth understanding of the fast dynamics on electrowetting and liquid penetration through GCNM will be an interesting research subject^41^,^42^.

Most previous reports in this field are limited to investigations of the planar movement of liquid droplets^43^. However, the proposed method of the electrowetting of graphene-coated meshes can realize three-dimensional movements of liquid droplets through adjustments of the applied electric voltage and by switching the location of the electric fields, leading to a variety of applications, such as active flow control, microfluidic devices and oil/water separation. Such functionally antagonistic active control devices can also facilitate new opportunities in electroactive flow control systems that operate in a manner analogous to electrical switches and transistors in electronic circuits.

## Methods

### Preparation and characterization of GCNM

A pure nickel mesh (Nilaco Corporation, 99.5%, opening width: 150 μm, wire diameter: 100 μm) was purchased and cut into 10 mm × 20 mm pieces. Graphene coatings were formed on the Ni meshes using a CVD method. The Ni meshes were heated to 1,000 °C under an Ar flow (1,000 sccm) at ambient pressure levels (see the Supplementary Note 1). After 10 min of annealing, a reaction gas containing a mixture of CH_4_ (80 sccm) and H_2_ (100 sccm) was flowed for 10 min to grow graphene on the Ni catalytic meshes. Finally, the samples were rapidly cooled to room temperature.

To investigate the continuity and integration of the graphene coating, the removal of the Ni mesh was done in each case with an additional etching step. The etching process involved simply soaking the samples in an HCl solution of 3 M for 24 h (see the Supplementary Note 2). The surface morphologies of the graphene meshes were observed by field emission SEM (Nova NanoSEM230). The samples were characterized by a dispersive Raman Microscope (Aramis, Horiba), equipped with a × 50 microscope objective lens and a 514 nm laser. The samples were analysed by multi-purpose an X-Ray photoelectron spectroscopy (Sigma Probe, Thermo VG Scientific).

### Electrical stimulation of water droplets

GCNM and copper tape (10 mm × 20 mm) were used for negative and positive electrodes, respectively. They were placed at a distance of 4 mm and connected to an external power source, a TREK COR-A-TROL (model 610B). The water droplet was placed on the GCNM electrode and an electric field was produced by applying a certain amount of voltage (0–5.5 kV) to the electrodes. In each experiment, a gradual increase from 0 V to the targeted voltage was required to ensure that the water droplet received steady stimulation. Droplets of KOH solution (0.1 M) and an ionic liquid (1-Butyl-3-methylimidazolium dicyanamide) were also used in the experiment.

### Measurement of contact angle

The CA of liquid droplets on the GCNM was measured using a SEO Phoenix CA system (Surface Electro Optics, Korea), equipped with an automatic dispenser and a CCD (charge coupled device) camera. Water droplets of 5 μl were generated by the automatic dispenser of the system. Images of the water droplets were captured by the CCD camera. The water CA of each sample was measured at four different positions, and the average and deviation values were presented. All measurements were done in an air environment at room temperature.

### Data availability

The data that support the findings of this study are available from the corresponding author upon request.

## Additional information

**How to cite this article:** Tabassian, R. *et al*. Graphene-coated meshes for electroactive flow control devices utilizing two antagonistic functions of repellency and permeability. *Nat. Commun.* **7**, 13345 doi: 10.1038/ncomms13345 (2016).

**Publisher's note:** Springer Nature remains neutral with regard to jurisdictional claims in published maps and institutional affiliations.

## Supplementary Material

Supplementary Information Supplementary Figures 1-11 and Supplementary Notes 1-6

Supplementary Movie 1 Reversible actuation of water droplet penetrating GCNM under electric field. As shown in Fig. 4c, one droplet (5μl) was placed on the mesh, while another droplet of the same size was positioned underneath the mesh. A Cu tape electrode was located above the mesh. By applying an electric field up to 13.75 kV/cm, the droplet underneath the mesh moved to the upper droplet through the mesh, and the fused droplets moved toward the Cu electrode. When the applied voltage was decreased to 0 kV, the droplets returned to their original shapes.

Supplementary Movie 2 Irreversible actuation of water droplets between two layered GCNM electrodes. We designed a novel electrode system with two water droplets and two GCNM electrodes, with one droplet per electrode, respectively, as shown in Fig. 5b. When an electrical field of 5 kV/cm was applied to the electrode system, the lower water droplet on the Cu electrode began stretching toward the GCNM, while the shape of the upper water droplet on the GCNM did not change in its shape. However, when the lower water droplet came into contact with the GCNM electrode at 5 kV/cm, the upper droplet abruptly passed through the GCNM mesh, as shown in Fig. 5b, due to the attractive and cohesive forces between the water drops. The electric field within the gap between the mesh and top of the droplet is greatly magnified, which cause the droplet at the bottom to touch the mesh on the upper size under a relatively small electric field. When the applied electric field was turned off, the merged water drop maintains this state without returning to the original states of the individual drops, thus showing an irreversible actuation mode.

Supplementary Movie 3 Locomotion of water droplets on multi-stage GCNM electrodes. A series of parallel mesh electrodes were aligned in the vertical direction, and water droplets were placed on each mesh. When an electrical field was applied to the first and second GCNM electrodes, the water droplet between them was actuated upward and suddenly pulled down the droplet placed on the upper electrode. The actuation mechanism is identical to that discussed in Fig. 5b. The highest water droplet merged into the second water droplet after passing through the first GCNM. Switching the location of the applied voltage between the two mesh electrodes sequentially caused the movement of water droplets from the top mesh to the bottom mesh electrodes, as shown in Fig. 5c.

Supplementary Movie 4 Electroactive flow switching device based on GCNM. A GCNM electrode and a Cu electrode were installed inside a rectangular flow channel standing vertically. In the absence of an electrical field, water was contained above the GCNM electrode without passing through the mesh due to the hydrophobic repellency of the mesh itself. When the electrical field was increased beyond a critical voltage, the contained water passed through the GCNM. This is the first demonstration of an electroactive flow switch based on graphene-coated metal meshes.
