# Supplementary material for: Graphene-coated meshes for electroactive flow control devices utilizing two antagonistic functions of repellency and permeability
Source: Nat Commun. 2016 Oct 31;7:13345. doi: 10.1038/ncomms13345 (PMC5095590; doi:10.1038/ncomms13345)
Supplement: Supplementary Information — Supplementary Figures 1-11 and Supplementary Notes 1-6 [file ncomms13345-s1.pdf]

Supplementary Figures

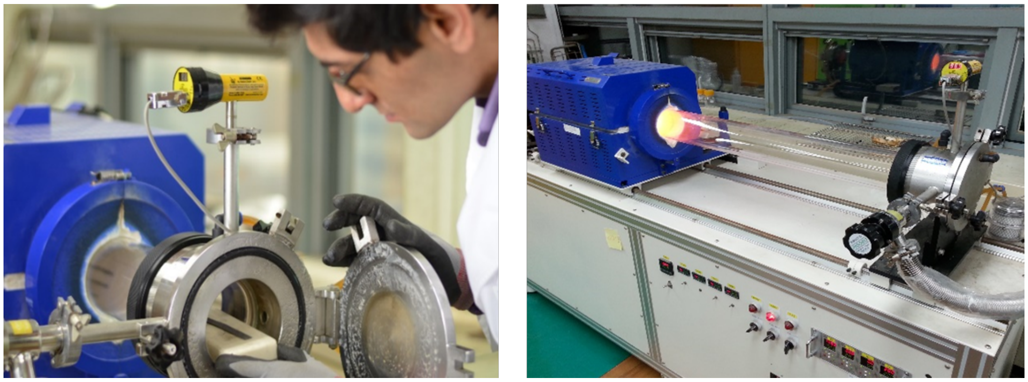

Supplementary Figure 1. CVD equipment used to coat graphene onto metal meshes.

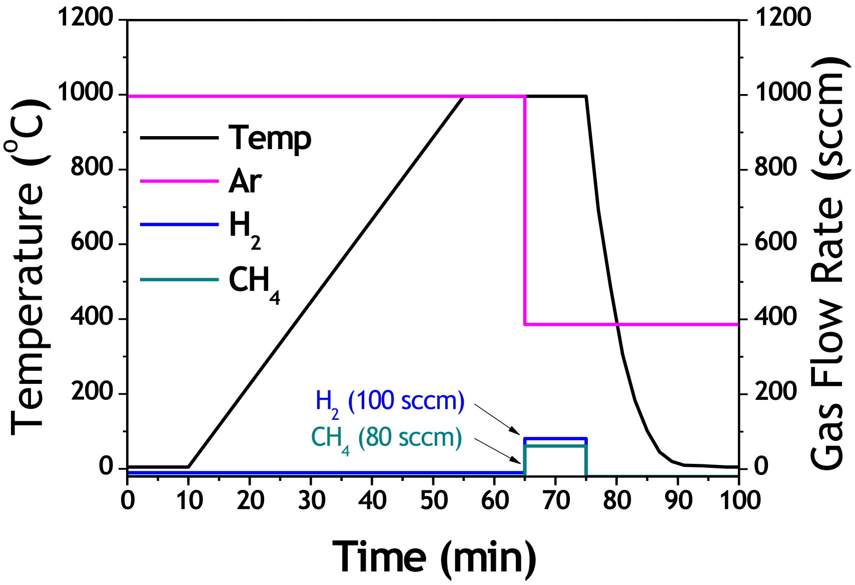

Supplementary Figure 2. CVD process diagram of graphene coating process onto a Ni mesh.

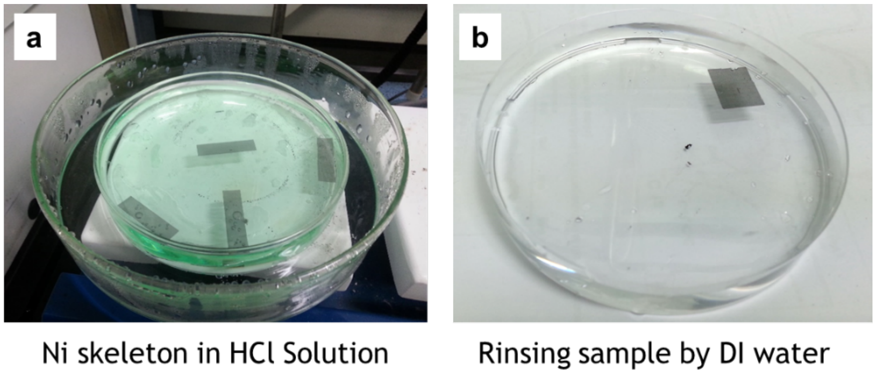

Supplementary Figure 3. Etching process of nickel skeleton.

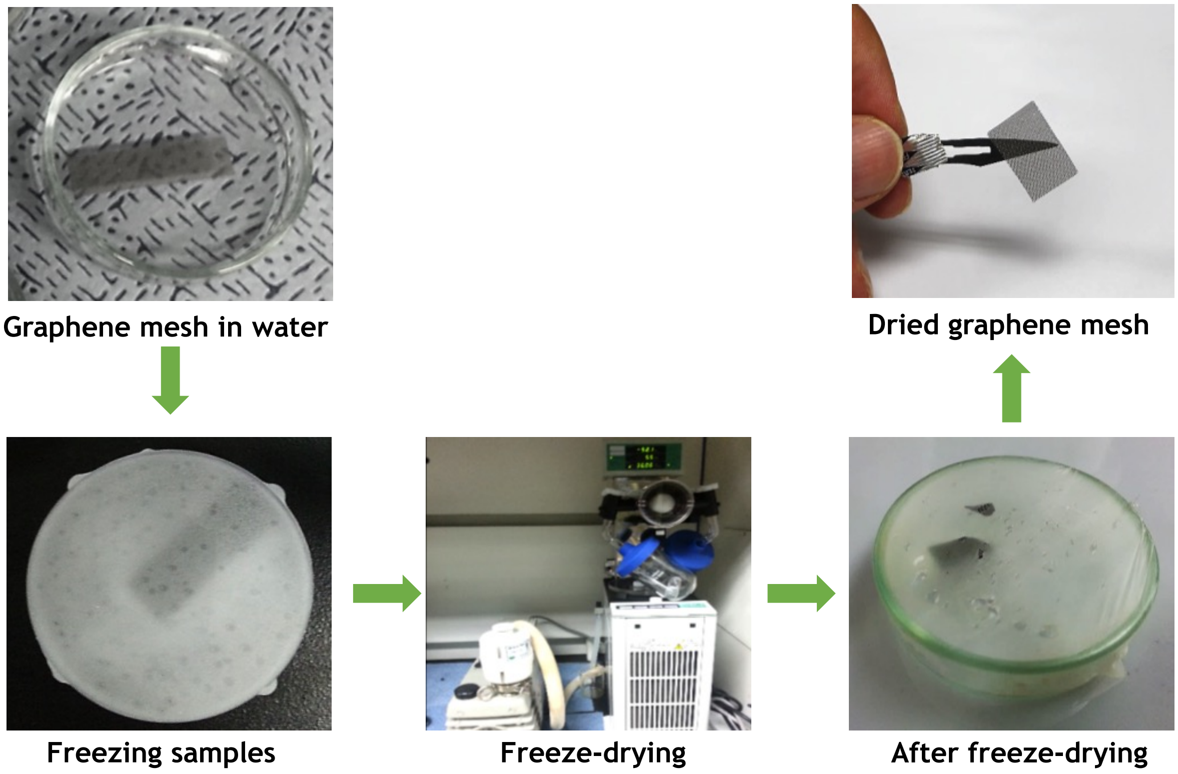

Supplementary Figure 4. Freeze-drying process of graphene mesh.

Bare Ni Mesh	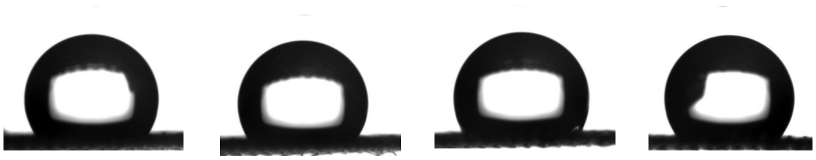 118.8 ° 118.3 ° 117.6 ° 119.3 °	Average 118.5 °
Graphene-coated Ni Mesh	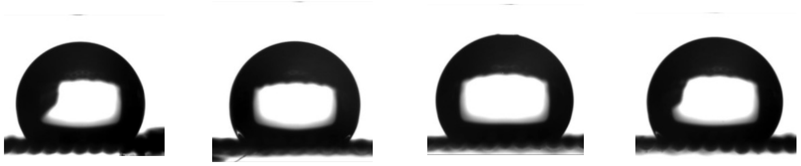 118.9 ° 120.2 ° 119.8 ° 117.7 °	Average 119.1 °

Supplementary Figure 5. Measurement of contact angle at four different points of the meshes. wetting transparency observed in metal meshes before and after graphene coating process.

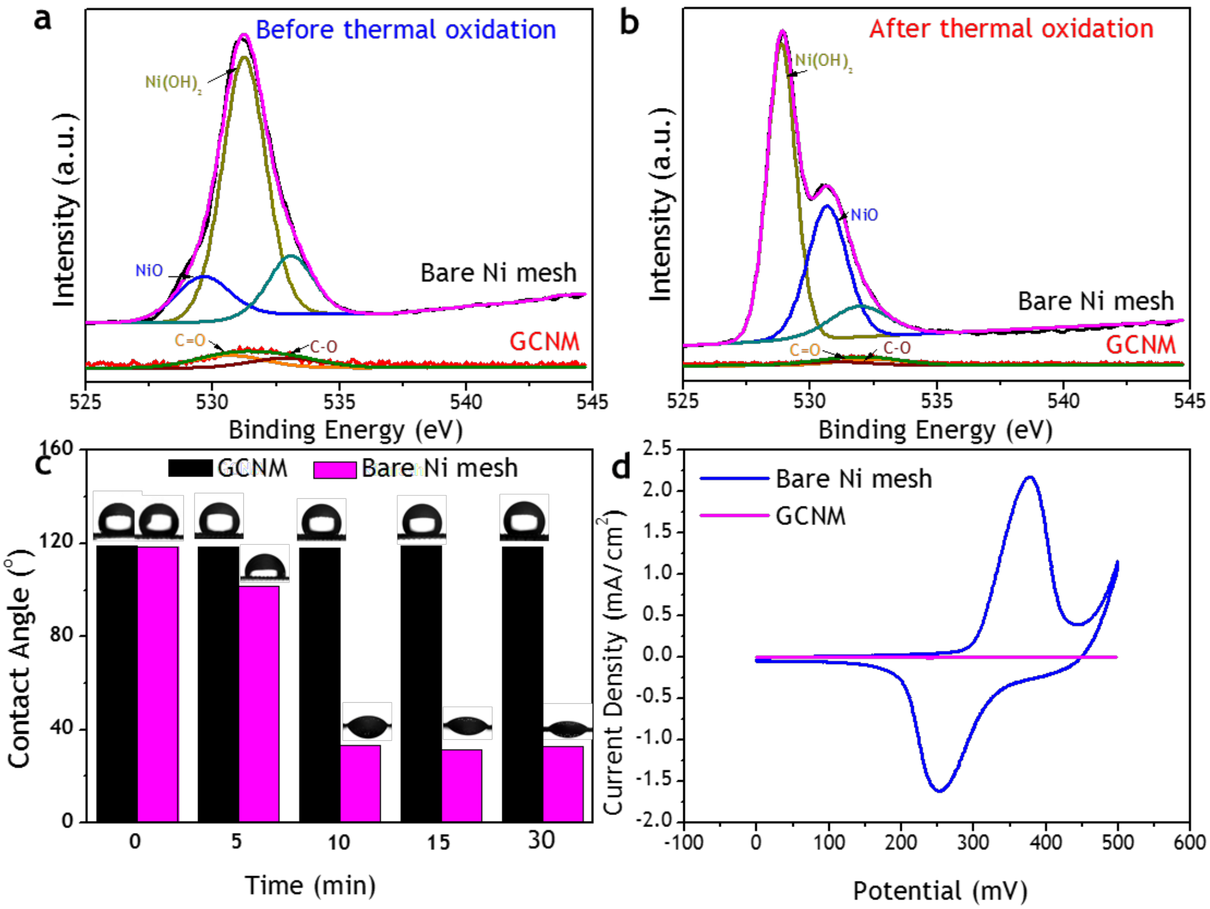

Supplementary Figure 6. Protective effect of graphene coating against oxidation and corrosion. (**a**) XPS spectra of bare nickel mesh and GCNM before thermal oxidation. (**b**) XPS spectra of bare nickel mesh and GCNM after thermal oxidation in 400°C for 4 hours. (**c**) Contact angles of bare nickel mesh and GCNM according to thermal oxidation time. (**d**) Electrochemical responses of bare nickel mesh and GCNM as a working electrode during cyclic voltammetry measurement.

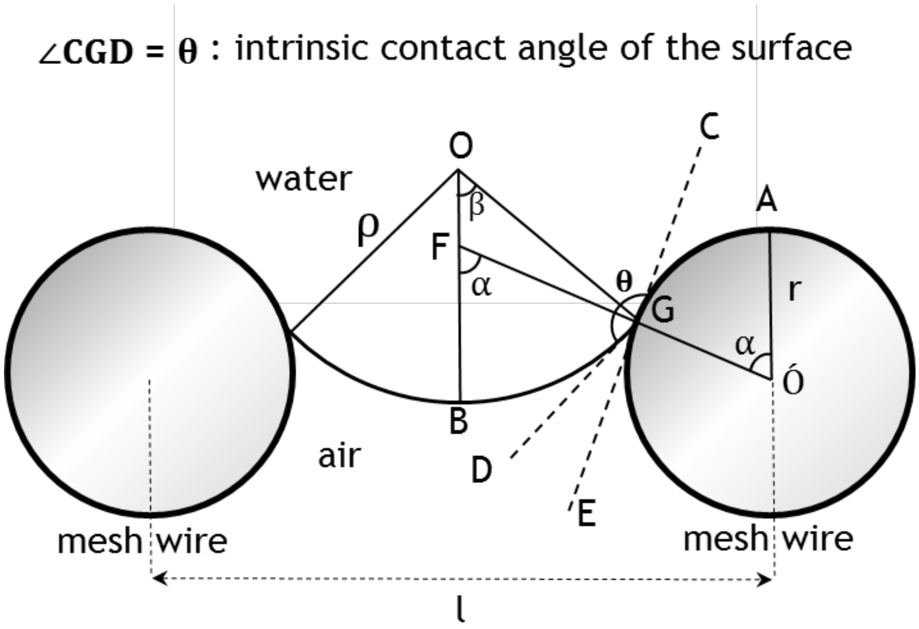

Supplementary Figure 7. Cross-sectional view of water profile on single hole of a mesh

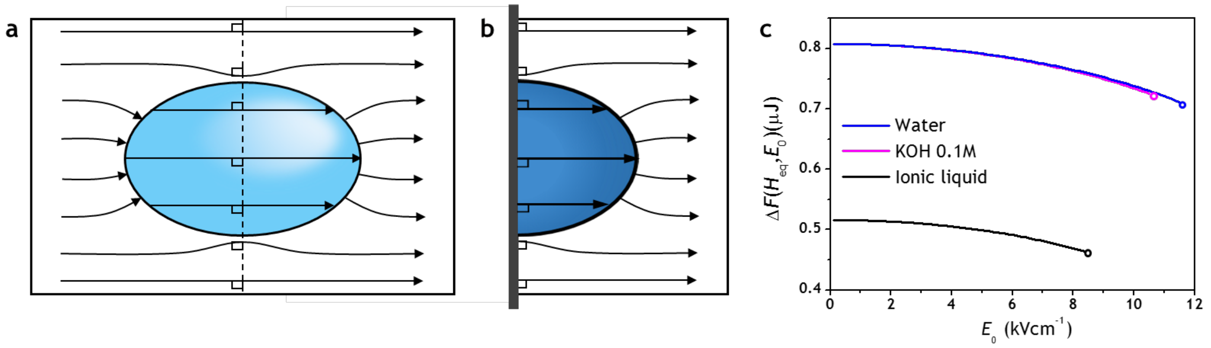

Supplementary Figure 8. (a) electric field distribution for the full ellipsoidal dielectric inclusion. (b) electric field distribution for the droplet when CA=90°. (c) droplet insertion free energy as a function of electric field. Beyond the threshold value, the free energy drops significantly and droplet height changes abruptly (refer to the last section in Supplementary Information for more details).

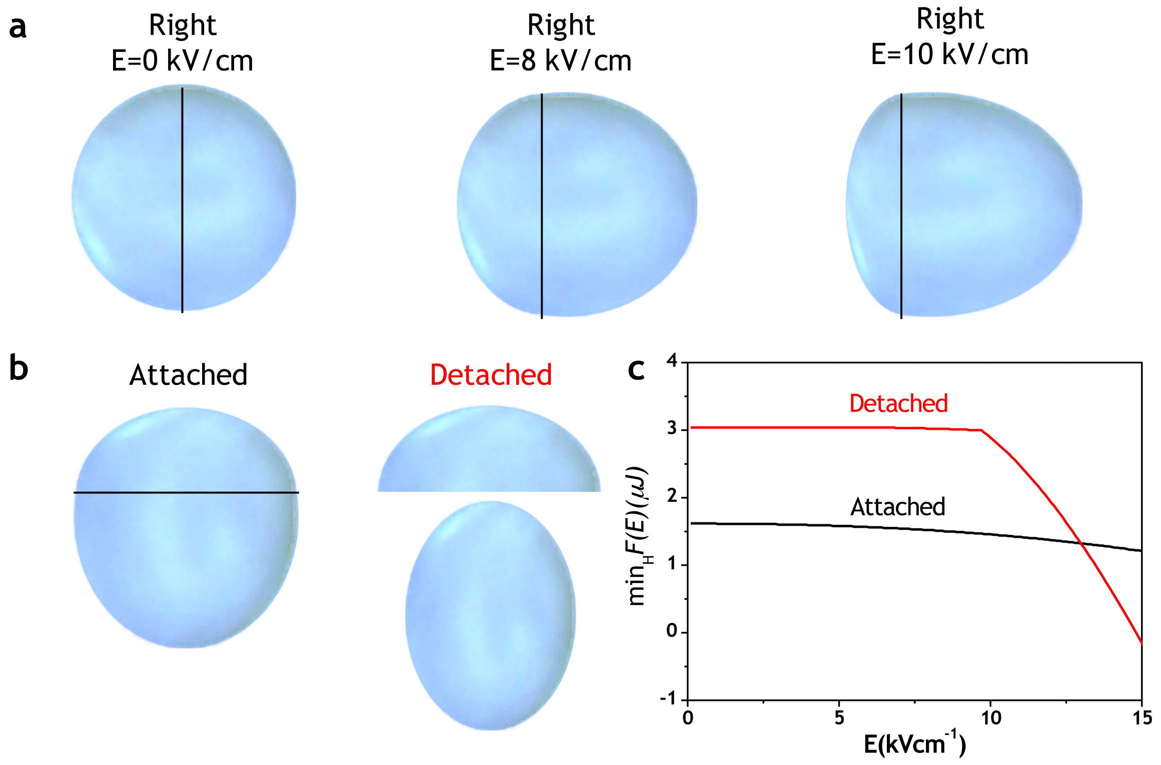

Supplementary Figure 9. (a) Predicted droplet shape change when electric field is applied at the right side. The volume of droplet at each side is 5 μl. This agrees qualitatively well with experiments (**Fig. 4d**) using the same volume. (b) Two states with the bottom droplet attached and detached. When the free energy associated in the detached state is lower than that in the attached state, the irreversible permeation of the droplet is initiated, as shown in **Fig. 4c**. (c) The calculated free energy as a function of the applied electric field, when the electric field distribution is formed for the droplet with CA=90°.

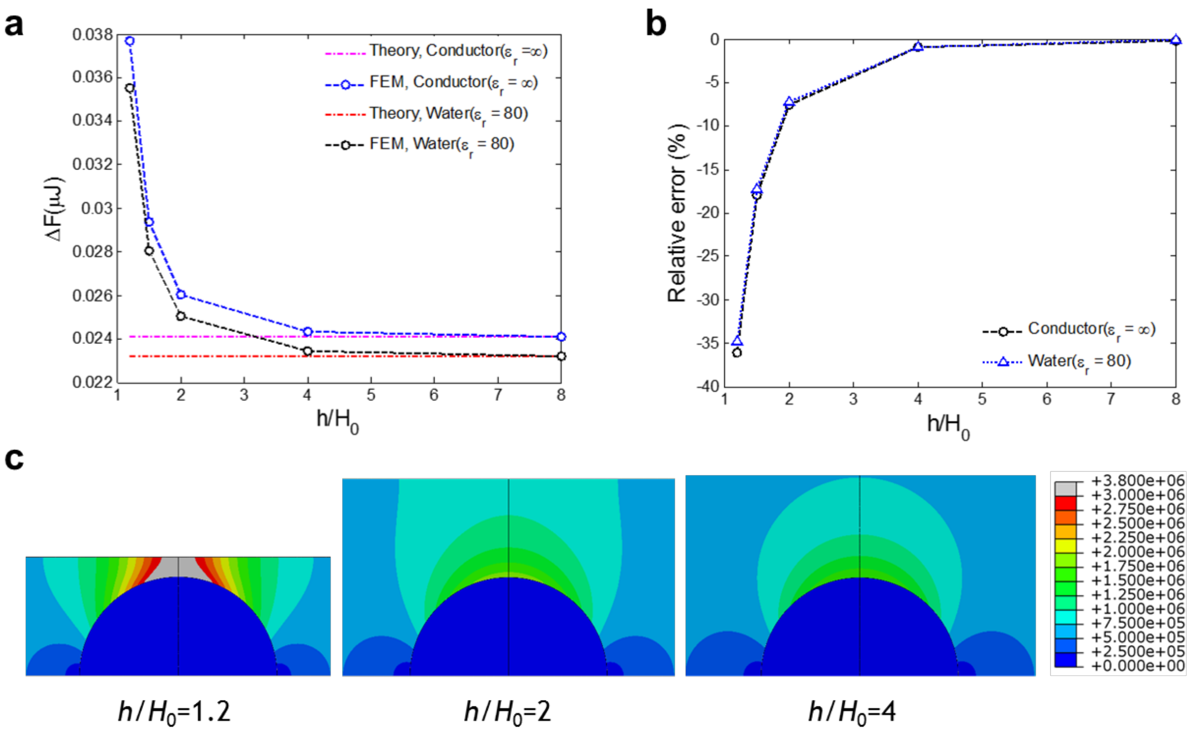

Supplementary Figure 10. (a) Comparison between the analytic solution for the infinite half space and the numerical solution as a function of h/H_0 for the hemi sphere droplet with a volume of 5 μl under a 6 kVcm^{-1} electric field. Due to the high relative permittivity of water, the water droplet solution is similar to a conducting droplet solution. (b) Relative error between analytical and numerical solutions. The relative error is less than 2% until h/H_0=4, which demonstrates the applicability of the analytic solution for the interpretation of the change in the droplet height in **Fig. 3**. The relative error becomes very high when $h/H_0 < 2$ due to the high electric field concentration near the gap between the droplet and the top electrode. (c) The distribution of the electric field magnitude in the vicinity of the droplet. The distribution is nearly identical to the theoretical solution at h/H_0=4, while a non-negligible deviation is found at h/H_0=2. When

h/H_0=1.2, a highly concentrated electric field profile is formed in the gap between the droplet and the top electrode.

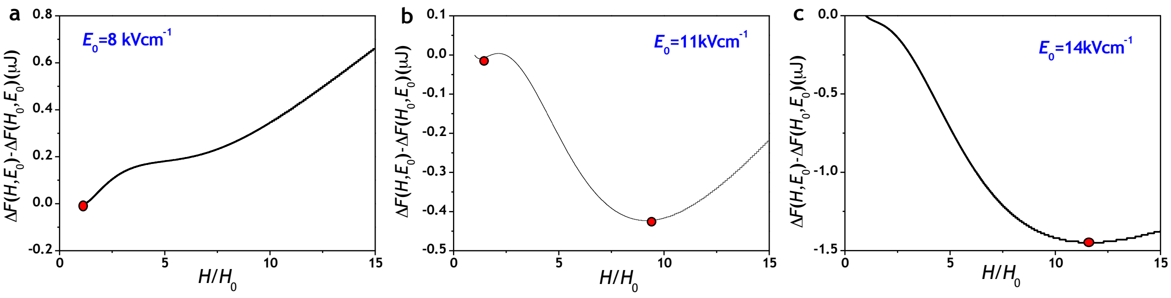

Supplementary Figure 11. Free energy as a function of the droplet height for a 5μl droplet at various applied electric fields. There is a single minimum at small H/H_0 when the electric field is small, while there are two minima at small and large H/H_0 points at intermediate electric fields. Beyond the threshold electric field, the minima at the small H/H_0 disappears and only another minimum at the large H/H_0 remains. Hence, below the threshold electric field, the droplet height follows a continuous change along the minimum at small H/H_0, after which the droplet height experiences a sudden discontinuous jump at the threshold electric field.

Supplementary Notes

Supplementary Note 1. Graphene coating on metal meshes

Nickel meshes were cut into the proper sizes and put into a CVD furnace as shown in **Supplementary Fig. 1**. The furnace was 40 cm long and could freely move along a 150 cm quartz tube with a diameter of 100 mm. The CVD method included the four steps of heating, annealing, growing, and cooling. First, the samples were heated to 1,000 ˚C under an Ar flow (1000 sccm) and at ambient pressure. After reaching the target reaction temperature, in order to clean the surface of the Ni mesh and remove the oxide layer, the samples were thermally annealed in this condition for 10 min. The Ar flow was then decreased to 400 sccm and a reaction gas mixture containing H_2 (100 sccm) and CH_4 (80 sccm) was flowed into the quartz tube to grow the graphene. After growing for 10 minutes, the H_2 and CH_4 injections were stopped while Ar continued to flow at the same rate (400 sccm). The furnace was then turned off and the samples were moved to another part of the quartz tube for rapid cooling of the samples to room temperature. After cooling, the Ar flow was also stopped and the samples were collected. The CVD process diagram is shown in **Supplementary Fig. 2**.

Supplementary Note 2. Etching process

In order to investigate the quality and integrity of the graphene coating, it was necessary to remove the nickel skeleton by an etching process. Without the Ni mesh skeleton, we were able to observe whether or not the graphene mesh maintained its shape. During the etching process, the GCNM was soaked in an HCl solution (3M) for 24 hours. To expedite the reaction, etching was done at

80˚C. During the process of etching out the nickel skeleton, the color of the solution changed to green and the hollow graphene mesh floated to the surface of the solution, as shown in **Supplementary Fig. 3a**. To ensure the complete etching of the Ni skeleton, a permanent magnet was used to test the samples. Because nickel is a ferromagnetic material, if the nickel was not fully etched it would be attracted to the magnet. After etching the nickel, the remaining graphene mesh was rinsed with DI water to remove the residual solution (**Supplementary Fig. 3b**).

To dry the graphene mesh after the etching process, a freeze-drying technique was utilized. This is the best way to retain the shape of the graphene during the drying step. First, the samples were frozen while they were in water. The pressure was then reduced to 14 Pa by a freeze-drying machine to evaporate the frozen water directly from the solid phase to the gas phase. Finally, the samples were collected while they maintained their shape, showing a fine mesh structure. The freeze-drying process steps are shown in **Supplementary Fig. 4**. In addition, CA measurements were taken of the mesh before and after the graphene coating process, which demonstrated the wetting transparency of the coated graphene, as shown in **Supplementary Fig. 5**.

Supplementary Note 3. Wetting states on the mesh and permeation through the mesh

The wettability of rough surfaces containing topographical features is generally described by two well-known models, Wenzel and Cassie models. In the Wenzel model, it is assumed that the liquid wets all surface topographies. Therefore, the actual solid/liquid interface is higher than apparent projected surface that is considered as the flat surface. In the Cassie model, liquid does not wet all portions of the porous surface, and some parts of the pores remain intact. As a result, the air trapped

in the pores increases the liquid/air interface and decreases the solid/liquid interface, which changes the wetting state of the surface.

When a drop is placed on a particular rough surface, either Cassie state or Wenzel state can occur depending on the sizes and shapes of the protrusions on the surface as well as the intensity of the external forces acting on the drop, like gravity and electrostatic forces. Even for a surface in the Cassie state, it is possible to change the wetting state to the Wenzel state by applying an external load and pushing the liquid into the pores on the surface. The Cassie state on the mesh can be defined by the Laplace equation. As shown in **Supplementary Fig. 7**, the angle α defines the position of three-phase interface (solid/liquid/air) on the surface of cylindrical wires. By changing the position of the three-phase interface, the water needs to change its surface curvature in order to create an intrinsic contact angle (*θ*) with the round surface of the cylindrical wires. The Laplace equation expresses the pressure produced by the liquid/air interface in terms of the free surface curvature;

$$\Delta P = \sigma_{\mathrm{la}}(\frac{1}{\rho_1} + \frac{1}{\rho_2}) = \frac{2\sigma_{\mathrm{la}}}{\rho}$$ (Supplementary Equation 1)

where σ_{la} is the surface tension of the liquid-air interface, and ρ_1 and ρ_2 are principal radii of curvature. With the assumption of square holes and straight wires, we assume that $\rho_1 = \rho_2 \approx \rho$. The radius of curvature, ρ, can be expressed in terms of α and θ.

$$2\rho \sin\beta = l - 2r \sin\alpha$$ (Supplementary Equation 2)

It is clear that $\angle\mathrm{GFB} = \angle\mathrm{A\acute{O}G} = \alpha$, $\angle\mathrm{DGE} = \angle\mathrm{FGO} = \pi - \theta$, and in FGO triangle $\angle\mathrm{GFB} = \angle\mathrm{FOG} + \angle\mathrm{OGF}$. Hence, $\beta + \pi - \theta = \alpha$ or $\beta = \alpha + \theta - \pi$ is valid. Substituting β in Supplementary Equation (2) yields

$$\rho = \frac{l - 2r \sin\alpha}{2\sin(\alpha + \theta - \pi)} = \frac{l - 2r \sin\alpha}{-2\sin(\alpha + \theta)}$$ (Supplementary Equation 3)

And thus,

$$\Delta P = \frac{-4\sigma_{\mathrm{la}}\sin(\alpha+\theta)}{l-2r\sin\alpha}. \qquad \text{(Supplementary Equation 4)}$$

Permeation occurs when the Laplace pressure ΔP by surface tension, which provides the pinning force, becomes smaller than the Maxwell stress induced by electric field at the apex of the droplet (point **B** in **Supplementary Fig. 7**). If we approximate the water profile near the apex as a conducting half prolate ellipsoid of eccentricity e, the intensified electric field at the apex under the applied electric field E is given by

$$E_{\mathrm{apex}} = \frac{2e^3}{(1-e^2)\left(\ln\frac{1+e}{1-e} - 2e\right)}E \qquad \text{(Supplementary Equation 5)}$$

For the perfect spherical shape with $e = 0$, the intensifying factor become 3, i.e., $E_{\mathrm{apex}} = 3E$. We note that the expression is exact for the conducting half prolate ellipsoid on a flat electrode. Due to very high relative permittivity of water, 80, it is reasonable to approximate the apex electric field near the water droplet using the solution for a conducting droplet. However, because we are considering a protruding water profile through a mesh, Supplementary Equation 5 can only be regarded as a first-order approximation. The actual electric field profile is very complex due to the presence of the conducting mesh near the water profile and cannot be solved analytically. Because E_{apex} is normal to the water surface, the Maxwell stress is given simply as $\frac{1}{2}\epsilon_0|E_{\mathrm{apex}}|^2$. As we increase the applied electric field, the curvature at the apex increases as the intensifying factor for the electric field rises; hence, the Laplace pressure also increases according to the curvature change. Beyond the threshold electric field, the apex point is sharpened significantly and becomes conical shape[39].

From the balance between the Laplace pressure and the Maxwell stress at the apex, or from the free energy consideration (refer to the next section for more details), the critical electric field is given by $\frac{E^2 r_0}{\sigma} \approx C$ where $r_0 \approx l/2$ is the radius of the water profile and C is a constant depending on the relative permittivities of the droplet and the surrounding medium. Hence, the threshold electric field scales as $E \sim \sqrt{\frac{\sigma}{\epsilon_0 r_0}}$. Because the threshold field for the larger droplet with $r_0 = H_0 = 1.34$ mm (5 μl) in **Fig. 3h** is around 10 kVcm^{-1}, the permeation threshold field for the mesh with $r_0 \approx l/2 = 130$ μm is estimated to be 33 kVcm^{-1}. Owing to such a high permeation threshold, permeation through the mesh is only observed when there exists another droplet on the other opposite side of the mesh (**Fig. 4b-c**), or when the droplet on the lower side becomes tall enough to touch the mesh electrode above (**Fig. 5b**).

Supplementary Note 4. Detailed theoretical analysis for electrical actuation of droplets

In the presence of the constant electric field $\mathbf{E_0}$, the droplet insertion free energy $\Delta F = \Delta F^{\mathrm{S}} + \Delta F^{\mathrm{e}}$ of the system can be described by two terms which refer to the surface energy contribution, ΔF^{S}, and the electrostatic contribution, ΔF^{e}. The surface energy contribution can be written as $\Delta F^{\mathrm{S}} = \sigma_{\mathrm{la}} S_{\mathrm{la}} + (\sigma_{\mathrm{ls}} - \sigma_{\mathrm{sa}}) S_{\mathrm{ls}}$ where σ_{la} is the surface tension of the liquid, σ_{ls} is the surface energy between the liquid and the substrate, and σ_{sa} is the surface energy of the substrate. S_{la} is the area of the droplet surface in contact with air, and S_{ls} is the area of the droplet in contact with the substrate. The electrostatic free energy change can be written as $\Delta F^{\mathrm{e}} = -\frac{1}{2} \int_{\mathrm{inside}} \mathbf{E_0} \cdot (\mathbf{D_l} - \epsilon_0 \epsilon_{\mathrm{r}}^{\mathrm{e}} \mathbf{E_l}) \mathrm{d}V$, where the volume integral is performed within the volume of the droplet, $\mathbf{D_l} = \epsilon_0 \epsilon_{\mathrm{r}}^{\mathrm{i}} \mathbf{E_l}$ refers to the electric displacement field inside the droplet, ϵ_0 refers to the permittivity of

the vacuum, $\epsilon_{\mathrm{r}}^{\mathrm{i}}, \epsilon_{\mathrm{r}}^{\mathrm{e}}$ refers to the relative permittivity inside and outside of the droplet, respectively, and $\mathbf{E_l}$ is the electric field inside the droplet. To summarize, the free energy of the system can be written as

$$\Delta F = \sigma_{\mathrm{la}} S_{\mathrm{la}} + (\sigma_{\mathrm{ls}} - \sigma_{\mathrm{sa}}) S_{\mathrm{ls}} - \frac{1}{2}\int_{\mathrm{inside}} \mathbf{E_0} \cdot (\mathbf{D_l} - \epsilon_0 \epsilon_{\mathrm{r}}^{\mathrm{e}} \mathbf{E_l}) \mathrm{d}V. \qquad \text{(Supplementary Equation 6)}$$

It is almost impossible to obtain the analytic solution for the ΔF for the general case, but we can obtain a analytical solution for the special case of θ_Y=90°, $\mathrm{i.e.}, \sigma_{\mathrm{lv}} \cos\theta_Y = \sigma_{\mathrm{sv}} - \sigma_{\mathrm{sl}}$, as the solution exist for the general ellipsoidal shape in free space. It is known that the electric field inside the ellipsoidal dielectric inclusion is uniform as depicted in **Supplementary Fig. 8a**, and an analytic solution is readily available. On the vertical plane of symmetry, the electric field inside and outside of the ellipsoid becomes orthogonal to the vertical plane. For a droplet sitting on a substrate at a contact angle of 90° as shown in **Supplementary Fig. 8b**, the electric field distribution becomes identical to the half that in the full ellipsoidal inclusion problem, as the electric field is orthogonal to the conducting electrode. Hence, the electrostatic free energy change can be obtained analytically. The electric field inside the prolate ellipsoid is known to be

$$\mathbf{E_l} = \frac{\epsilon_{\mathrm{r}}^{\mathrm{e}} \mathbf{E_0}}{(1-n)\epsilon_{\mathrm{r}}^{\mathrm{e}} + n\epsilon_{\mathrm{r}}^{\mathrm{i}}} \qquad \text{(Supplementary Equation 6)}$$

where geometrical factor n is given as a function of the eccentricity of the ellipsoid $e = \sqrt{1-\left(\frac{R}{H}\right)^2}$, as

$$n = \frac{1-e^2}{2e^3}\left(\ln\frac{1+e}{1-e} - 2e\right). \qquad \text{(Supplementary Equation 7)}$$

It can easily be shown that when R=H , $n = \frac{1}{3}$ and $\mathbf{E_l} = \frac{\epsilon_{\mathrm{r}}^{\mathrm{e}} \mathbf{E_0}}{2\epsilon_{\mathrm{r}}^{\mathrm{e}} + \epsilon_{\mathrm{r}}^{\mathrm{i}}}$ which are the well-known solutions for a spherical inclusion. For the special case of $\theta_Y = 90°$, the volume integral becomes a simple algebraic expression in proportion to the droplet volume, the surface area of the droplet

become half of the ellipsoidal area, and $\sigma_{ls} - \sigma_{\mathrm{sa}}$ becomes zero, which simplifies the equation significantly. Finally, the insertion free energy of the droplet with $\theta_Y = 90°$ can be written as follows:

$$\Delta F = \sigma_{\mathrm{la}}\left(\pi R^2\left(1 + \frac{H}{Re}\sin^{-1} e\right)\right) - \frac{1}{2}\cdot\frac{2}{3}\pi H R^2 \cdot E_0^2 \frac{\epsilon_0(\epsilon_{\mathrm{r}}^{\mathrm{i}}-1)}{(1-n)+n\epsilon_{\mathrm{r}}^{\mathrm{i}}} \qquad \text{(Supplementary Equation 8)}$$

Because the volume of the droplet, $V_0 = \frac{2}{3}\pi H R^2$, does not change under the presence of the electric field, we can express R as a function H, as $R = \sqrt{\frac{3V_0}{2\pi H}}$. Hence, the free energy becomes a function of two variables, i.e., $\Delta F(H, E_0)$. As discussed in the main text, at a given applied field E_0, the droplet height can be obtained from the local minimum of $\Delta F(H, E_0)$. We note that the free energy change is a monotonically decreasing function of the applied electric field E_0 (**Supplementary Fig. 8c**); hence, it is always favorable to have the droplet on the side under an arbitrary electric field. However, droplet locomotion through the mesh only occurs beyond a very high threshold electric field (around 33 $\mathrm{kVcm^{-1}}$) due to the presence of the energy barrier associated with the Laplace pressure, as explained in the previous section in the Supplementary Information.

Due to such a high threshold electric field, we study the permeation of the droplet by two different methods. First, we initiate the permeation when the droplet on the lower side becomes tall enough to touch the mesh electrode above (**Fig. 5b**) under a relatively small electric field. Because of the small gap between the mesh plate and the droplet at the lower side, the electric field within the gap can be magnified by a few times. When the width of the gap is d_{gap}, the height of the droplet is H and applied electric field is E (defined by the applied voltage divided by the distance between electrode), the average electric field in the gap can be estimated to be $\frac{H}{d_{\mathrm{gap}}}E$ because we can

assume that the electric field within the droplet is very small compared to the electric field outside due to the high relative permittivity of water (ϵ_{r} of water is 80).

Second, we initiate the permeation by placing another droplet at the other opposite side of the mesh, as shown in **Fig. 4b-c**. We perform back-of-the-envelope calculations on the droplet shape change and the permeation threshold. For simplicity, we do not consider the effect of gravity. The droplet shape change can be understood by computing the minimum free energy geometry when the electric field is applied onto the right side of the mesh, as depicted in **Supplementary Fig. 9a**. To solve the problem analytically, we consider a case in which contact angles of both droplets are 90° in the absence of an applied electric field. In this case, the droplet shape on the left side in the absence of an electric field must be a spherical cap to minimize the surface energy, and the droplet shape on the right side under an electric field is a half prolate ellipsoid to minimize the free energy. The total free energy can be written as

$$\Delta F_{\mathrm{a}} = \sigma_{\mathrm{la}} S_1 + \sigma_{\mathrm{la}} S_2 - \frac{1}{2} \cdot V_2 \cdot E_0^2 \frac{\epsilon_0(\epsilon_{\mathrm{r}}^{\mathrm{i}}-1)}{(1-n_2)+n_2\epsilon_{\mathrm{r}}^{\mathrm{i}}} \qquad \text{(Supplementary Equation 9)}$$

with the constraints, $V_1 + V_2 = \frac{4}{3}\pi R^3$ and R as a constant. S_1 and S_2 (V_1 and V_2) are the surface area (volume) of the droplets on the left and right side. When H_1 and H_2 are the corresponding heights of the droplets on the left and right side, we obtain $S_1 = \pi(R^2 + H_1^2)$, $S_2 = \pi R^2\left(1 + \frac{H_2}{Re_2}\sin^{-1} e_2\right)$, $e_2 = \sqrt{1 - \frac{R^2}{H_2^2}}$, $V_1 = \frac{\pi H_1}{6}(3R^2 + H_1^2)$, $V_2 = \frac{2}{3}\pi H_2 R^2$, and $n_2 = \frac{1-e_2^2}{2e_2^3}(\ln\frac{1+e_2}{1-e_2} - 2e_2)$. Under a given electric field, the droplet shape is determined by $H_{1,\mathrm{eq}}$ and $H_{2,\mathrm{eq}}$, which minimizes the free energy ΔF_{a} in Eq. (S9), as depicted in **Supplementary Fig. 9a**. In order to estimate the irreversible droplet shape change, we then compute the free energy ΔF_d when the droplet of volume V_2 on the nonzero electric field side

is detached, as depicted in **Supplementary Fig. 9b**. While only a fraction of the bottom droplet is detached in actual experiments, we consider a state with the entire bottom droplet detached to simplify the calculation. The free energy of the detached state can be written as

$$\Delta F_{\mathrm{d}} = \sigma_{\mathrm{la}} S_1 + \sigma_{\mathrm{la}} \widetilde{S_2} - \frac{1}{2} \cdot V_2 \cdot E_0^2 \frac{\epsilon_0 \left(\epsilon_{\mathrm{r}}^{\mathrm{i}} - 1\right)}{(1 - \widetilde{n_2}) + \widetilde{n_2} \epsilon_{\mathrm{r}}^{\mathrm{i}}} \qquad \text{(Supplementary Equation 10)}$$

where $\widetilde{S_2}$ and $\widetilde{n_2}$ are the surface area and the geometrical factor for the full prolate ellipsoid with volume V_2. By assuming that the maximum major axis to minor axis ratio is 4 (similar to the ratio of the distance between the electrode and the radius of the droplet), we can obtain ΔF_{a} and ΔF_{d} as functions of electric field. When the electric field is small, the attached state has lower free energy due to the smaller surface area, while the detached state has lower free energy under high electric field because it can lower the volume term significantly by accommodating higher geometrical factor, $\widetilde{n_2}$. Crossover occurs when the applied electric field is approximately 13 kVcm^{-1}, which is similar to the experimental value (**Fig. 5a**).

Supplementary Note 5. Effect of the droplet size on the electric field distribution and electrostatic energy

While the distance between the electrodes is finite in experiments, we use the analytic solution for the infinite half space to describe the energetics of the droplet. Here, we compare the analytic solution with the numerically computed the electrostatic energy of a hemisphere droplet as a function of h/H_0 where h is the distance between the electrodes and H_0 is the radius of the droplet, as shown in **Supplementary Fig. 10**. The ABAQUS package is used for the numerical calculation. For the hemisphere, the electrostatic energy (the second term in Eq (3)) is determined by

$$\Delta E_{\mathrm{electrostatic}} = \frac{1}{2} \cdot \frac{2}{3} \pi H_0^3 \cdot E_0^2 \frac{\epsilon_0(\epsilon_{\mathrm{r}}^{\mathrm{i}} - 1)}{\frac{2}{3} + \frac{1}{3}\epsilon_{\mathrm{r}}^{\mathrm{i}}} \quad \text{(Supplementary Equation 11)}$$

because the geometrically factor is $n = 1/3$ when the eccentricity is zero. We find that the analytic solution shows a very good match in the range $h/H_0 > 4$, while a significant relative difference is found at $\frac{h}{H_0} < 2$ due to the highly intensified electric field in the small gap between the droplet and the top electrode.

Supplementary Note 6. A detailed analysis on the divergence of droplet height in terms of free energy

In this section, we provide a more thorough analysis of the free energy given in Eq. (3), and the divergence of the droplet height. Under a given electric field E_0, the minimum in the free energy is determined by the balance between the surface term (the first term) and the volume term (the second term). With an increase in eccentricity of the droplet, the surface term monotonically increases, while the magnitude of the volume term increases relatively quickly in the small eccentricity range but becomes saturated in the large eccentricity range because the lower bound of the geometrical factor n is zero. Depending on the magnitude of the electric field, E_0, we can have three characteristic curves, as shown in **Supplementary Fig. 11**. In the small range of e, there is a single minimum at a small droplet height, while in the intermediate range of e, there are two minima at small and large droplet heights. Beyond the threshold e, there remains only a single minimum at a large droplet height and the minimum at a small droplet height disappears. A more detailed analysis of the free energy curve near the small minimum can be found in the literature, though the study does not discuss the number of free energy minima in the droplet height range. Below the threshold electric field, the droplet height changes continuously along the free energy

minima at a small droplet height. Above the threshold electric field, the droplet height experiences a discontinuous jump to another minimum at a high droplet height. As discussed in the previous sections in the Supplementary Information, the threshold electric field scales as $E \sim \sqrt{\frac{\sigma}{\epsilon_0 r_0}}$. Of course, all of the discussion is correct only if we can approximate the droplet shape as a part of an ellipsoid. However, in experiments, it has been found that the shape of droplet tip is sharpened beyond the threshold electric field, resembling a cone (which is referred as a Taylor cone). In our experiments, we also found that the droplet is split into smaller pieces under a very high electric field.
